# Effects of a novel toll-like receptor 4 antagonist IAXO-102 in a murine model of chemotherapy-induced gastrointestinal toxicity

**DOI:** 10.1007/s00280-022-04463-x

**Published:** 2022-08-12

**Authors:** Janine S. Y. Tam, Elise E. Crame, Aurelia S. Elz, Janet K. Coller, Anthony Wignall, Clive A. Prestidge, Joanne M. Bowen

**Affiliations:** 1 Discipline of Physiology, School of Biomedicine, University of Adelaide, Adelaide, SA 5005 Australia; 2 Clinical and Health Sciences, University of South Australia, Adelaide, SA Australia; 3 Discipline of Pharmacology, School of Biomedicine, University of Adelaide, Adelaide, SA Australia; 4 ARC Centre of Excellence in Convergent Bio-Nano Science and Technology, VIC Parkville, Australia

**Keywords:** Toll-like receptor 4 (TLR4), TLR4 antagonist, Gastrointestinal mucositis, CPT-11, Tumour, MC-38 cells

## Abstract

**Introduction:**

Gastrointestinal mucositis (GIM) is a side effect of high-dose irinotecan (CPT-11), causing debilitating symptoms that are often poorly managed. The role of TLR4 in the development of GIM has been clearly demonstrated. We, therefore, aimed to investigate the potential of the TLR4 antagonist, IAXO-102, to attenuate gastrointestinal inflammation as well as supress tumour activity in a colorectal-tumour-bearing mouse model of GIM induced by CPT-11.

**Methods:**

24 C57BL/6 mice received a vehicle, daily i.p. IAXO-102 (3 mg/kg), i.p. CPT-11 (270 mg/kg) or a combination of CPT-11 and IAXO-102. GIM was assessed using validated toxicity markers. At 72 h, colon and tumour tissue were collected and examined for histopathological changes and RT-PCR for genes of interest; TLR4, MD-2, CD-14, MyD88, IL-6, IL-6R, CXCL2, CXCR1, and CXCR2.

**Results:**

IAXO-102 prevented diarrhoea in mice treated with CPT-11. Tumour volume in IAXO-102-treated mice was lower compared to vehicle at 48 h (*P* < 0.05). There were no differences observed in colon and tumour weights between the treatment groups. Mice who received the combination treatment had improved tissue injury score (*P* < 0.05) in the colon but did not show any improvements in cell proliferation or apoptotic rate. Expression of all genes was similar across all treatment groups in the tumour (*P* > 0.05). In the colon, there was a difference in transcript expression in vehicle vs. IAXO-102 (*P* < 0.05) and CPT-11 vs. combination (*P* < 0.01) in MD-2 and IL-6R, respectively.

**Conclusion:**

IAXO-102 was able to attenuate symptomatic parameters of GIM induced by CPT-11 as well as reduce tissue injury in the colon. However, there was no effect on cell proliferation and apoptosis. As such, TLR4 activation plays a partial role in GIM development but further research is required to understand the specific inflammatory signals underpinning tissue-level changes.

## Introduction

Gastrointestinal mucositis (GIM) is a difficult to manage complication of cancer treatment characterised by inflammation of the mucosa of the intestinal tract that leads to immunological, functional, and structural changes [1]. It is a common side effect of high-dose irinotecan (CPT-11) and remains one of the most debilitating side effects of cancer treatment despite decades of research. GIM has also been known to cause other symptoms such as pain, nausea, vomiting and diarrhoea [2]. These symptoms significantly reduce patient quality of life, as well as survival, as GIM can negatively impact tolerance of chemotherapy which leads to discontinuation or de-escalation of treatment.

The pathobiology of acute intestinal inflammation as seen in GIM following CPT-11 has been linked to the activation of innate immune receptor TLR4. In GIM, TLR4 activation upregulates pro-inflammatory cytokines TNF-α and IL-6 [1]. This occurs via a downstream signalling pathway whereby CPT-11 causes direct injury to the intestinal epithelial cells, allowing the luminal antigens to enter the lamina propria. Antigen-derived lipopolysaccharides (LPS), or endotoxins, then activate TLR4 expressed on the basal membrane of epithelial cells and mucosa-associated immune cells [3]. Subsequently, these interactions lead to inflammation and eventual ulceration. Ulceration then leads to enhanced translocation of luminal contents and increases the risk of bacteraemia in immunocompromised patients [3]. There has been previous research examining the role of TLR4 on the development of CPT-11-induced mucositis. However, to date there has been no consistency in the role of TLR4 in the development of CPT-11-induced GIM [4, 5]. A study by Boeing et al. reported that the colon of wild-type mice treated with CPT-11 displayed an increase in histoarchitecture loss, inflammatory infiltrate and the presence of cryptitis compared to the colon of vehicle treated mice [6]. Mice that are germ-free, thus lacking LPS signals are also protected from CPT-11 GI injury [7]. However, due to limitations of genetically modified animals in research translation, research efforts are now targeted at tailoring methods of inhibiting TLR4 pharmacologically to confirm its role in GIM.

Previous experiments have also shown that TLR4 expression by tumour cells can be a contributing factor that promotes tumour cell proliferation, survival, migration, and metastasis [8]. Research has shown that tumours activated the suppression of T-cell and natural killer cell activity, but when TLR4 was inhibited, this tumour-mediated suppression of T-cell and natural killer cells was prevented, which delayed tumour growth and increased survival of the tumour-bearing mice [9]. Another study showed LPS stimulation of the TLR4/MD-2 complex can activate downstream signalling pathways that promotes the adhesiveness and metastatic capacity of colorectal cancer (CRC) cells [10]. These findings have shown the impact TLR4 has in CRC progression. While TLR4 activation can increase tumour growth and immunosuppression, it can also promote anti-tumour activity. For example, a study has shown that TLR4 expressed on dendritic cells plays an important role in promoting anti-tumour immune responses following chemotherapy [11].

Any treatment that modifies TLR4 signalling may have protective effects for the intestine while also increasing anti-tumour activity during chemotherapy. However, there has yet to be a specific TLR4 antagonist used in a tumour-bearing preclinical model to investigate the impact on GIM and tumour growth simultaneously. IAXO is a highly specific ligand that interferes selectively with the TLR4 and its co-receptors MD-2 and CD-14. IAXO-102 has been investigated in experimental studies of abdominal aortic aneurysms to date displaying its ability to inhibit TLR4 and subsequent downstream effects in an inflammatory disease [12]. This study therefore aimed to investigate the potential of IAXO-102 to attenuate gastrointestinal inflammation as well as suppress tumour activity in a colorectal tumour-bearing mouse model of GIM induced by CPT-11.

## Methods

### Animal model and ethics

The study was approved by the University of Adelaide Animal Ethics Committee (M-2021–033) and complied with the National Health and Research Council Australia (Australia) Code of Practice for Animal Care in Research and Training (2013) [13]. Mice were group housed in ventilated cages (*n* = 3–6 mice/cage) with a 12 h light/dark cycle, while food and water were provided ad libitum.

### Experimental design

**Fig. 1:**
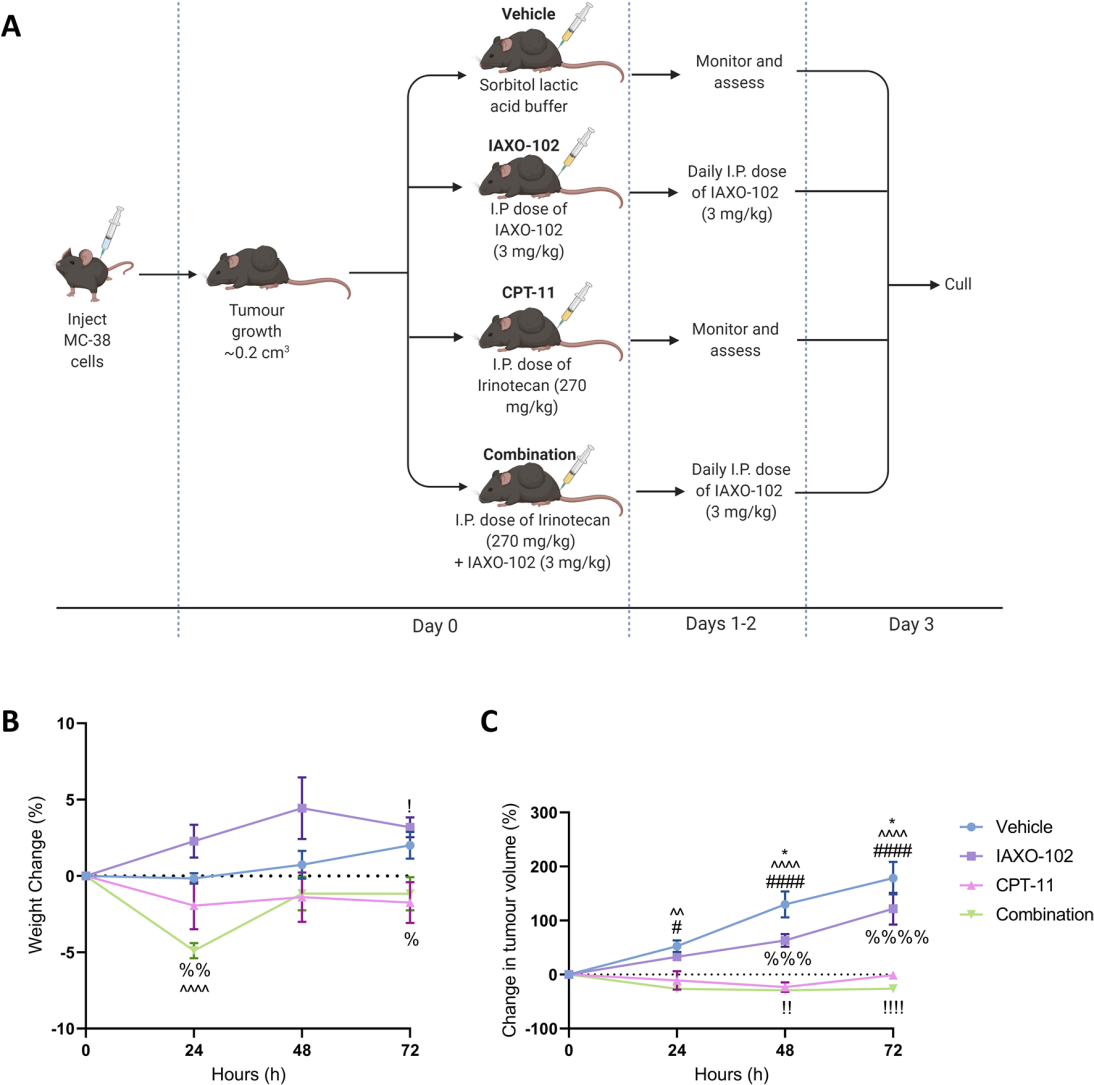
Experimental timeline and toxicity assessment. **A** Experimental timeline showing the sequence of events and treatment timepoints. **B** Percentage change in weight over 72 h. Data displayed as a mean ± standard error of the mean (SEM) percentage weight change from baseline (0 h), *n* = 6 per group. **C** Tumour volume over 72 h. Data displayed as a mean ± SEM percentage change in tumour volume from baseline (0 h), *n* = 6 per group. Symbols indicate statistical significance: vehicle group vs. IAXO-102 group: * *P* < 0.05; vehicle group vs. CPT-11 group: # *P* < 0.05, #### *P* < 0.0001; vehicle group vs. combination group: ^^ *P* < 0.01, ^^^^ *P* < 0.0001; IAXO-102 group vs. CPT-11 group: ! *P* < 0.05, !! *P* < 0.01, !!!! *P* < 0.0001; IAXO-102 group vs. combination group: % *P* < 0.05, %% *P* < 0.01, %%% *P* < 0.001, %%%% *P* < 0.0001

All mice were on a C57BL/6 background. Female and male mice (*n*_total_ = 24) weighing between 15 and 25 g (6–13 weeks of age) were bred in the University of Adelaide Laboratory Animal Service (SA, Australia). Mice were subcutaneously transplanted in the right flank with 2 × 10^6^ cells/mL MC-38 cells, a murine colon adenocarcinoma cell line derived from C57BL/6 mice as previously published in Secombe et al. [14]. MC-38 cells were kindly provided by Associate Professor Michele Teng of the Cancer Immunoregulation and Immunotherapy Laboratory, QIMR Berghofer Medical Research Institute, Australia. When tumour growth reached approximately 0.2 cm^3^ the mice were treated with either of the following: 3 days of daily 3 mg/kg intraperitoneal (i.p.) dose of the TLR4 antagonist IAXO-102 (MedChemExpress, USA) in a diluent of 10% EtOH, 40% PEG400, 5% Tween-80 and 45% saline; a single 270 mg/kg i.p. dose of CPT-11 (kindly provided by Pharmacia/Pfizer, USA) prepared in a sorbitol/lactic acid buffer (45 mg/mL sorbitol/0.9 mg/mL lactic acid; pH 3.4; Sigma-Aldrich, USA); the combination of CPT-11 and IAXO-102; or sorbitol/lactic acid buffer only (vehicle mice) which acted as the control group. Equivalence in body weight and tumour size on the day of treatment was confirmed by ANOVA. Mice were randomly assigned to treatment groups and culled by cervical dislocation at 72 h after being anaesthetised using inhalation isoflurane (1 L/min O_2_ with 4% isofluorane). The study timeline is shown in Fig. 1A.

### Assessment of intestinal toxicity

Mice were weighed daily to track weight loss/gain. All mice were monitored twice daily for the presence of diarrhoea (scored as present or absent) and other toxicity parameters: ruffled coat, dehydration, hunched posture, rectal bleeding, and reluctance to move. Mice were killed if they displayed ≥ 15% weight loss or significant distress and deterioration, in compliance with animal study ethical requirements.

### Tissue preparation

The entire gastrointestinal tract from pyloric sphincter to rectum was dissected and flushed with chilled 1 × phosphate buffered saline (PBS, pH 7.4, ThermoFisher Scientific, USA) to remove contents. The large intestine was weighed immediately after resection. Tumours were removed and weighed after skin and fat were dissected off. All weights were then represented as relative to body weight on the day of cull. Samples of colon (1 cm in length) and tumour were collected and (i) drop-fixed using 10% neutral buffered saline for processing and embedding into paraffin wax, or (ii) snap frozen in liquid nitrogen and stored at − 80 °C for molecular analyses.

### Histopathologic analysis

Haematoxylin and eosin (H&E) staining was performed on 5 μm sections of colon cut on a rotary microtome and mounted onto glass Menzel-Gläser Superfrost microscope slides (ThermoFisher Scientific). Slides were scanned using the NanoZoomer (Hamamatsu Photonics, Japan) and assessed with NanoZoomer Digital Pathology software.view2 (NDP.view2, Version 2.7.39) (Hamamatsu Photonics). The occurrence of eight histological criteria in the colon was examined to generate a total tissue injury score [15]. These criteria were disruption of brush border, architectural disruption, disruption of crypt cells, and infiltration of polymorphonuclear leukocytes cells, dilation of lymphatics and capillaries, oedema, reduction in goblet cell number and thickening of muscularis externa. Each parameter was scored as present = 1 or absent = 0 in a blinded fashion by two independent assessors (J.S.Y. Tam/A. Wignall). Concordance on all scores was confirmed between assessors.

### Immunohistochemistry assessment of cellular markers of apoptosis and proliferation

Immunohistochemistry (IHC) was carried out on 5 μm sections of colon and tumour, cut on a rotary microtome and mounted onto FLEX IHC microscope slides (Agilent, USA). IHC analysis was performed for Ki67 (Abcam; #ab16667), a marker of proliferation and caspase-3 (Abcam; #ab4051), a marker of apoptosis. Changes in both parameters are validated markers for altered tissue kinetics and an excellent way to assess the subclinical severity of toxicity [16]. IHC analysis was performed using Agilent reagents on an automated machine (AutostainerPlus, Agilent) following standard protocols supplied by the manufacturer. Briefly, sections were deparaffinised in xylene and rehydrated through graded ethanols before undergoing heat-mediated antigen retrieval using an EDTA/Tris buffer (0.37 g/L EDTA, 1.21 g/L Tris; pH 9.0). Retrieval buffer was preheated to 65 °C using the Dako PT LINK (pretreatment module; Agilent; #PT101). Slides were immersed in the buffer, and the temperature was raised to 97 °C for 20 min. After returning to 65 °C, slides were removed and placed in the Agilent AutostainerPlus (Agilent; #AS480) and stained following manufacturer's guidelines. Negative controls had the primary antibodies omitted. Slides were scanned using the NanoZoomer (Hamamatsu Photonics) and assessed with NDP.view2 software (Hamamatsu Photonics). Cell proliferation data were represented as the percentage of positively stained cells relative to total cells in the intestinal crypts. Apoptosis was quantified by counting the number of positively stained cells for 15 crypts and the data were presented as average positively stained cells per crypt. Only well-oriented, non-oblique crypts were included for analysis. A scoring system of percentage area of cells stained brown in the tissue: 0—25% = 0; 26–50% = 1; 51—75% = 2; 76–100% = 3 was used to analyse the tumour tissue stained with Ki67 and caspase-3. Two blinded investigators (J.S.Y Tam/A. Wignall) independently scored each stained section and mean score from both investigators were calculated. Concordance was confirmed between investigators on all scoring results.

### RT-PCR for markers of TLR4 signalling

RNA was isolated from snap frozen tumour and colonic tissue using the NucleoSpin® RNA Plus kit (Macherey–Nagel, Germany) following the manufacturer′s protocol. RNA was quantified using a Synergy™ Mx Monochromator-Based Multi-Mode Microplate Reader (BioTek, USA) and reverse transcribed using an iScript cDNA Synthesis Kit (Bio-Rad Laboratories, USA) according to the manufacturer′s protocol. cDNA was quantified using a Synergy™ Mx Monochromator-Based Multi-Mode Microplate Reader (BioTek) and diluted to a working concentration of 100 ng/μL in nuclease-free water. Expression of key markers of TLR4/MD-2 downstream signalling pathway were investigated. Primers for genes of interest were designed using web-based primer design programme, PRIMER 3 (v. 0.4.0) and manufactured by Sigma-Aldrich (Table 1). Amplified transcripts were detected by SYBR Green (Qiagen Pty Ltd., Australia) in a Rotor-Gene Q Series Rotary Cycler (Qiagen Pty Ltd.). All reactions were completed in triplicate including a non-template control to determine presence of contamination. The relative ratio of mRNA expression was calculated using 2^∆Ct^ method using β-actin as the normalising housekeeper gene [17]. β-actin has been shown to have stable expression levels across cell types and treatments [18].

**Table 1:** Mouse RT-PCR primer sequences designed by PRIMER 3 (v. 0.4.0)

TLR4	Forward: 5 ‘-CTC TGC CTT CAC TAC AGA GAC-3’ Reverse: 5’-TGG ATG ATG TTG GCA GCA ATG-3’
MD-2	Forward: 5 ‘-GTC CGA TGG TCT TCC TGG CGA GT-3’ Reverse: 5’-GCT TCT CAG ATT CAG TCA ATA TGG G-3’
CD-14	Forward: 5 ‘-GTC AGG AAC TCT GGC TTT GC-3’ Reverse: 5’-GGC TTT TAC CCA CTG AAC CA-3’
IL-6	Forward: 5 ‘-AGT TGC CTT CTT GGG ACT GA-3’ Reverse: 5’-TCC ACG ATT TCC CAG AGA AC-3’
IL-6R	Forward: 5 ‘-TGA ATG ATG ACC CCA GGC AC-3’ Reverse: 5’-ACA CCC ATC CGC TCT CTA CT-3’
CXCR2	Forward: 5 ‘-GCA GAG GAT GGC CTA GTC AG-3’ Reverse: 5’-TCC ACC TAC TCC CAT TCC TG-3’
CXCL1	Forward: 5 ‘-GGG TGA AGC CAC AAC AGA TT-3’ Reverse: 5’-GCA GAC CAG CAT AGT GAG CA-3’
CXCL2	Forward: 5 ‘-GCA GAG GAT GGC CTA GTC AG-3’ Reverse: 5’-TCC ACC TAC TCC CAT TCC TG-3’
β-actin	Forward: 5 ‘-CTC TTC CAG CCT TCC TTC CT-3’ Reverse: 5’-AGC ACT GTG TTG GCG TAC AG-3’

### Statistical analysis

Data was graphed and analysed using GraphPad Prism Software 9.0 (GraphPad® Software, San Diego, USA). A D'Agostino & Pearson normality tests were conducted to determine if data was parametric or non-parametric. A Kruskal–Wallis test with Dunn’s multiple comparisons test was performed on non-parametric data to compare between the treatment groups. A two-way ANOVA with Tukey's multiple comparisons test was performed on parametric data to compare between the treatment groups. Any data point that had a value more than 3 times the standard deviation from the mean was excluded as an outlier. *P* values of < 0.05 were considered statistically significant.

## Results

### Mice treated with IAXO-102 were protected from CPT-11-induced GIM symptom of diarrhoea

Weight loss following CPT-11 treatment was most severe at 24 h in the combination group (− 4.90% ± 1.22% vs baseline) (Fig. 1B). While the IAXO-102 group gained the most weight at 24 h (2.28% ± 2.65% vs baseline) and 48 h (4.43% ± 4.95 vs baseline). The weight loss in the combination group was different compared to the vehicle (*P* < 0.0001) at 24 h, and the IAXO-102 group at 24 h (*P* < 0.01) and 72 h (*P* < 0.05). While the CPT-11 group had a difference in weight compared to the IAXO-102 group at 72 h (*P* < 0.05).

CPT-11 caused diarrhoea in 50% of mice within 6 h of CPT-11 administration and 100% at 24 h (Table 2). However, IAXO-102 treatment attenuated diarrhoea of CPT-11 GIM as diarrhoea was prevented in mice in the combination group (Table 2). No diarrhoea was seen in any vehicle or IAXO-102 treated mice (data not presented).

**Table 2:** Toxicity symptoms over 72 h. Data presented as total number of animals (per time point). Toxicity parameters includes: ruffled coat, dehydration, hunched posture, rectal bleeding and reluctance to move

Toxicity symptoms	CPT-11 (number of animals)				Combination (number of animals)			
	6 h	24 h	48 h	72 h	6 h	24 h	48 h	72 h
Diarrhoea	5/6	6/6	0/6	0/6	0/6	0/6	0/6	0/6
Toxicity parameters	3/6	3/6	1/6	0/6	5/6	2/6	1/6	0/6

### IAXO slowed colorectal tumour growth

Tumours were measured daily and expressed as a change in volume from the day of CPT-11 injection. From 24 to 72 h, tumour volume of the vehicle group was higher compared to the CPT-11 group (24 h: *P* < 0.05; 48 and 72 h: *P* < 0.0001) and combination group (24 h: *P* < 0.01; 48 and 72 h: *P* < 0.0001) (Fig. 1C). Tumour volume of the IAXO-102 group was different at 48 h and 72 h compared to the vehicle group (48 h and 72 h: *P* < 0.05), the CPT-11 group (48 h: *P* < 0.05; 72 h: *P* < 0.05) and the combination group (48 h: *P* < 0.001; 72 h: *P* < 0.0001) (Fig. 1C). There were no differences in tumour volume of the CPT-11 and combination group from 24 to 72 h (Fig. 1C).

### There were no differences in colon and tumour weights of mice between treatment groups

**Fig. 2:**
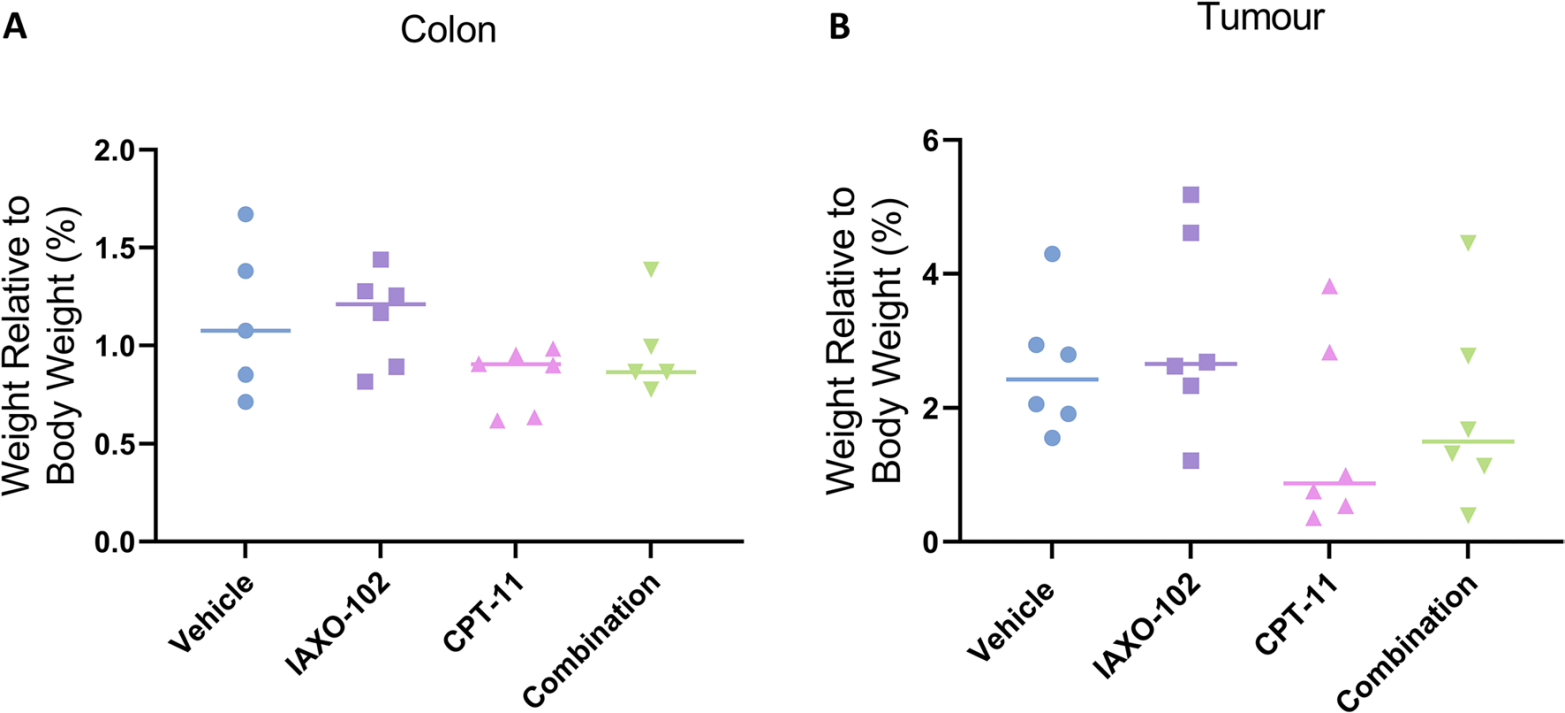
Organ wet weight of all treatment groups. **A** Colon wet weights. **B** Tumour wet weights. All data displayed as a percentage of weight relative to body weight and lines represent group median, *n* = 5–6 per group. Symbols indicate statistical significance: IAXO-102 vs. CPT-11: ! *P* < 0.05

There was no difference in colon wet weights between the treatment groups (Fig. 2A). There were also no differences observed in tumour weights between the treatment groups (Fig. 2B).

### IAXO–102 protects against CPT-11-induced colonic histopathology independent of cell death and turnover

**Fig. 3:**
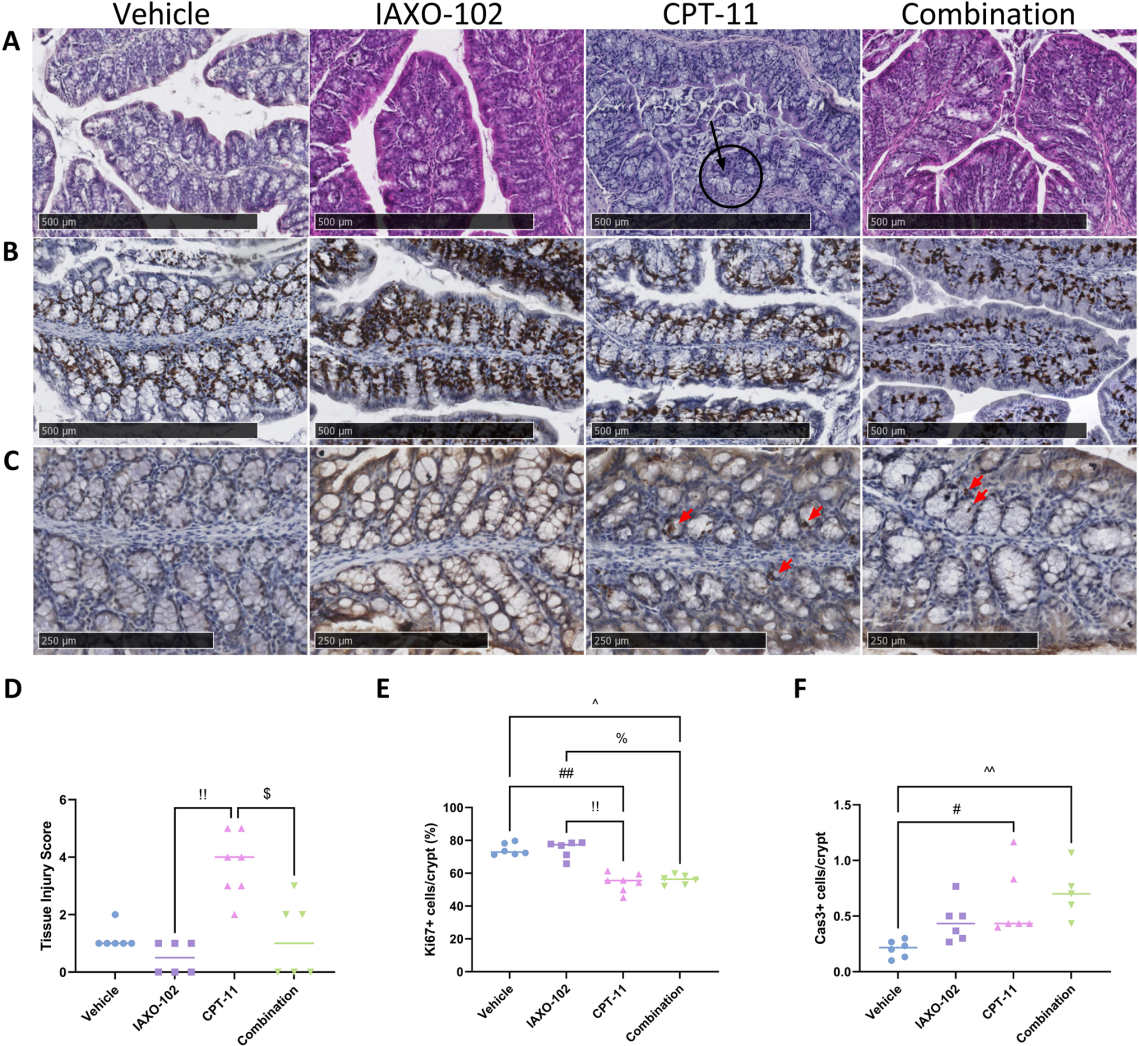
H&E and IHC staining results in the colon. **A** Representative H & E images showing epithelial disruption (black arrow) and inflammatory infiltrate (black circle). Scale bars, 500 µm. 40 × original magnification. **B** Representative immunostaining of Ki67 cells in colonic crypts. Proliferating cells are stained brown. Scale bars, 500 µm. 40 × original magnification. **C** Representative immunostaining of caspase-3 cells in colonic crypts. Apoptotic cells are stained brown (red arrow). Scale bars, 250 µm. 40 × original magnification. **D** Histopathological tissue injury scores in the colon of mice. Data presented as median, *n* = 6 per group. **E** Percentage of Ki67 positively stained cells in the colonic crypts. Data presented as median, *n* = 6 per group. **F** Number of caspase-3 positively stained cells in the colonic crypts. Data presented as median, *n* = 5–6 per group. Symbols indicate statistical significance: vehicle vs. CPT-11: # *P* < 0.05, ## *P* < 0.01; vehicle vs. combination: ^ *P* < 0.05, ^^ *P* < 0.01; IAXO-102 vs. CPT-11: !! *P* < 0.01; IAXO-102 vs. combination: % *P* < 0.05; CPT-11 vs. combination: $ *P* < 0.05

Representative H & E images (Fig. 3A) show minimal damage in vehicle, IAXO-102 and combination groups. CPT-11 treatment caused epithelial disruption (black arrow) and inflammatory infiltrate (black circle). Histopathological analysis (Fig. 3D) showed that combination treated mice were protected against CPT-11-induced mucosal tissue injury in the colon, with a lower histopathological score compared to the CPT-11 group (*P* < 0.05). The IAXO-102 group also had a difference in tissue injury score compared to the CPT-11 group (*P* < 0.01). There were no other differences observed between the groups.

Representative images show Ki67 positive cells (stained brown, Fig. 3B). Analysis of the Ki67 images (Fig. 3E) showed that the CPT-11 group had a decrease in proliferating cells compared to the vehicle and IAXO-102 groups (*P* < 0.01). The combination group also had a lower number of proliferating cells compared to the vehicle and IAXO-102 groups (*P* < 0.05). There were no differences observed between the other groups.

Representative images of caspase-3 positive cells in the colonic crypts are shown (red arrow, Fig. 3C). Analysis of the caspase-3 images (Fig. 3F) showed that the CPT-11 (*P* < 0.05) and combination (*P* < 0.01) groups had a higher apoptotic rate compared to the vehicle group. There were no other differences observed between the groups.

### Tumours in mice treated with CPT-11 had a higher apoptotic score compared to mice treated with vehicle

A scoring system of pertcentage area of cells stained brown in the tissue: 0 =  ≤ 25%; 1 = 26–50%; 2 = 51–75%; 3 ≥ 76% was used to analyse the tumour tissue stained for Ki67 and caspase-3.

**Fig. 4:**
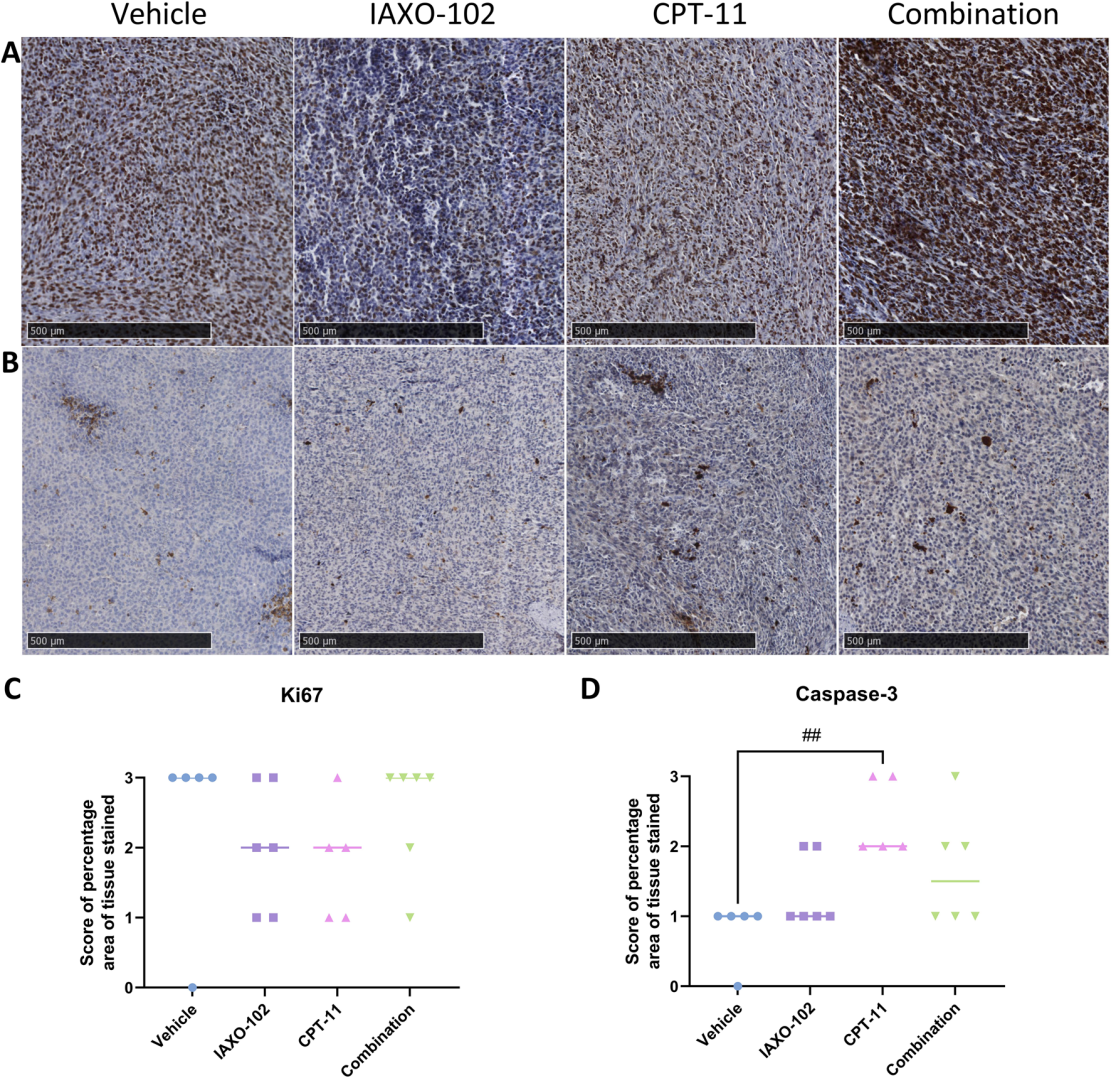
IHC staining results in the tumour. **A** Representative immunostaining of Ki67 cells in tumour tissue. Proliferating cells are stained brown. Scale bars, 500 µm. 40 × original magnification. **B** Representative immunostaining of caspase-3 cells in tumour tissue. Apoptotic cells are stained brown. Scale bars, 500 µm. 40 × original magnification. **C** Analysis and scoring of tumour tissue stained with Ki67. Data presented as median, *n* = 5–6 per group. **D** Analysis and scoring of tumour tissue stained with Caspase-3. Data presented as median, *n* = 5–6 per group. Symbols indicate statistical significance: vehicle vs. CPT-11: ## *P* < 0.01

Representative immunostaining images of proliferating cells (Ki67 positive cells stained brown) in tumour tissue (Fig. 4A) revealed no differences in scores for positively stained proliferating cells between the groups (Fig. 4C).

Representative immunostaining images of apoptotic cells (caspase-3 positive cells stained brown) in tumour tissue (Fig. 4B) revealed that the CPT-11 group had a higher score for positively stained apoptotic cells compared to the vehicle group (*P* < 0.01) (Fig. 4D). There were no other differences observed between the other groups (Fig. 4D).

### Effect of IAXO-102 treatment on gene expression in mouse colonic tissue

**Fig. 5:**
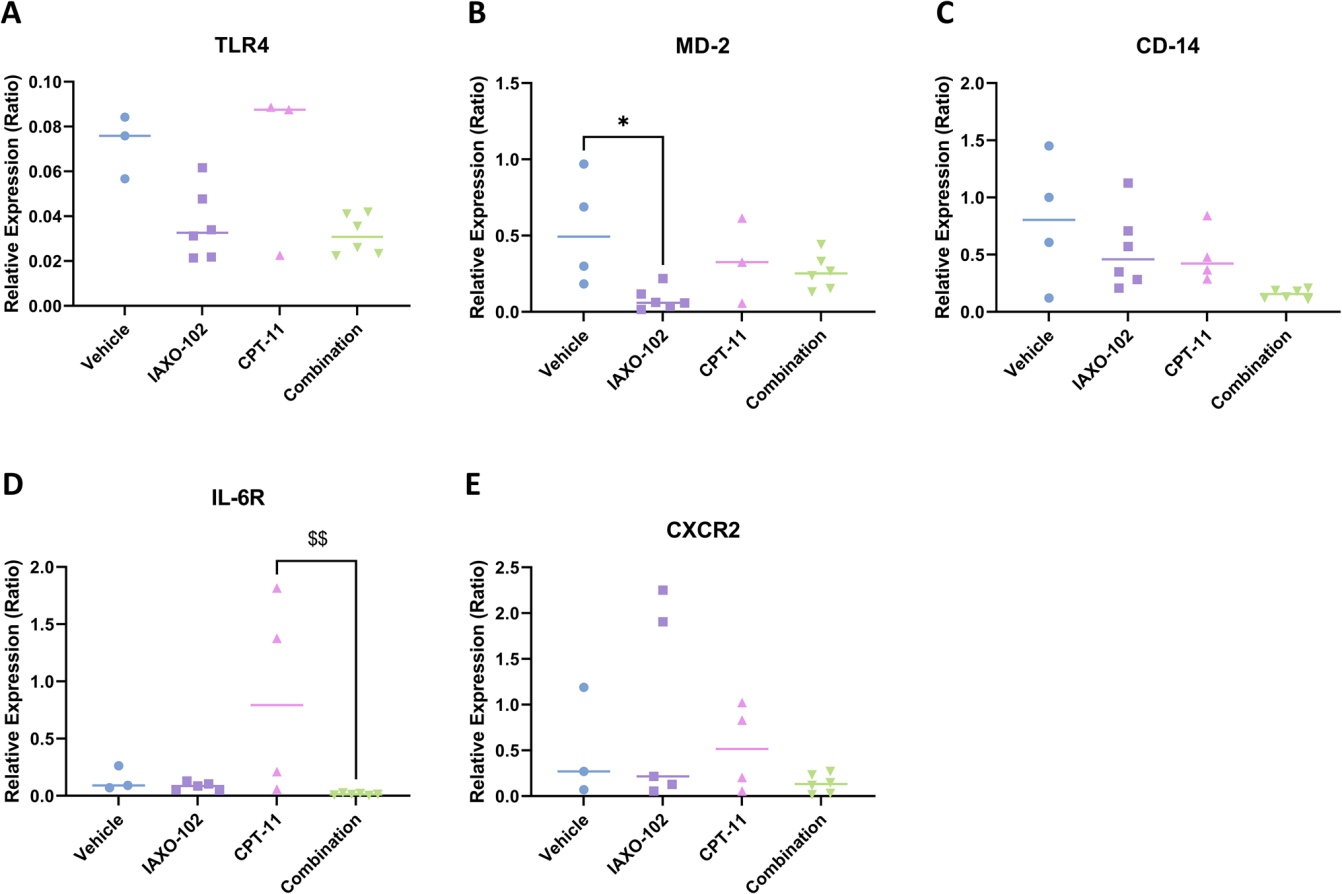
Transcript expression in the colon. **A** TLR4, **B** MD-2, **C** CD-14, **D** IL-6R, and **E** CXCR2 from colonic tissue relative to the housekeeper β-actin. Data are presented as median, *n* = 3–6 per group. Symbols indicate statistical significance: vehicle vs. IAXO-102: * *P* < 0.05; CPT-11 vs. combination: $$ *P* < 0.01

There was no change in transcript levels between treatment groups for **TLR4** (Fig. 5A), **CD-14** (Fig. 5C), and **CXCR2** (Fig. 5E). A difference was observed in **MD-2** transcript levels between vehicle and IAXO-102 groups (P < 0.05); no other differences were observed between the groups (Fig. 5B). A difference was also observed in **IL-6R** transcript levels between CPT-11 and combination groups (P < 0.01); no other differences were observed between the groups (Fig. 5D). The transcript expression of IL-6, CXCL1 and CXCR1 were investigated in the colon but there was no expression in any of the treatment groups.

### Effect of IAXO-102 treatment on gene expression in mouse tumour tissue

**Fig. 6:**
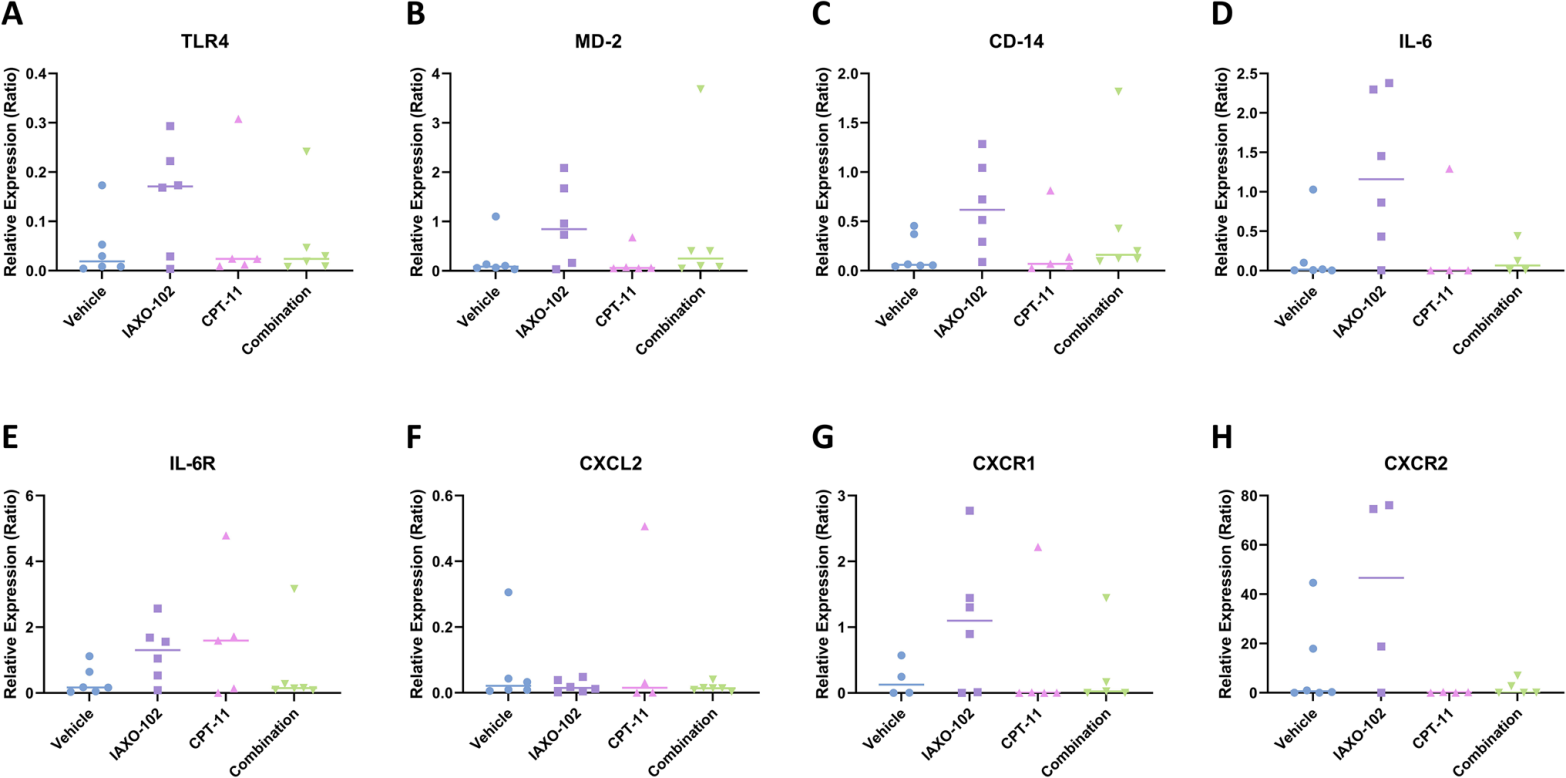
Transcript expression in the tumour. **A** TLR4, **B** MD-2, **C** CD-14, **D** IL-6, **E** IL-6R, **F** CXCL2, **G** CXCR1 and **H** CXCR2 from tumour tissue relative to the housekeeper β-actin. Data are presented as median, *n* = 4–6 per group

Levels of transcript expression in the tumour tissue of all the groups were also analysed. There was no change in transcript levels across any groups in any of the genes of interest; **TLR4** (Fig. 6A); **MD-2** (Fig. 6B); **CD-14** (Fig. 6C); **IL-6** (Fig. 6D); **IL-6R** (Fig. 6E); **CXCL2** (Fig. 6F); **CXCR1** (Fig. 6G); **CXCR2** (Fig. 6H).

## Discussion

Inflammation of the mucosa of the intestinal tract during cancer treatment is known as GIM and is most severe during high-dose chemotherapy. TLR4 signalling has been strongly implicated in the development and treatment of CRC and GIM through its regulation of inflammation. This study explored how interruption of TLR4 signalling using a pharmacological intervention modulates these outcomes. To exclude any influence of sex on the results, we also did a sub-analysis and found that male and female mice had equivalent body weight change, diarrhoea incidence and tumour growth.

It was found that the TLR4 antagonist IAXO-102 was able to prevent diarrhoea in mice treated with CPT-11. The dose and schedule of IAXO-102 was equivalent to previous work that found protection against inflammation was associated with downregulation of TLR4 protein expression [12]. Diarrhoea reduction was associated with improved histopathological scores, indicating retention of colonic morphology and subsequent function. Work by others have shown similar protective effects using non-specific TLR4 antagonists. For example, a study by Fakiha et al. reported that amitriptyline was able to prevent CPT-11-induced diarrhoea and colonic apoptosis in rats but did not see any protective effects in histological architecture in the intestinal tract [19]. Although not using a TLR4 antagonist, a study by Wardill et al. found that TLR4 knock-out mice were protected against CPT-11-induced mucosal tissue injury in the small intestine and also displayed a reduction in CPT-11-induced diarrhoea [5]. Another study has also shown that pharmacological inhibition of TLR4 was able to reduce disease activity and prevent morphological damage in an inflamed colon [20]. In contrast, a study using tumour-bearing rats reported that naloxone did not improve GIM following CPT-11 treatment. The naloxone also did not improve any weight loss and even increased tumour growth in the rats [21]. Collectively this provides evidence that targeting TLR4 signalling interferes with development of GIM and warrants further investigation.

The mechanisms by which TLR4 inhibition protects colonic tissue and prevents diarrhoea was then further investigated using well-established tissue markers. The typical markers of CPT-11-induced injury, apoptosis and reduced proliferation of crypt epithelial cells [16], were not significantly affected by IAXO-102. The lack of measurable changes may be due to the kinetics of cell death and halting of the cell cycle following chemotherapy exposure. Previous studies have shown that apoptosis may be an early indicator of intestinal damage with rates peaking at 6 h after administration of CPT-11 [5, 19]. Although slightly slower than apoptosis, halting of the cell cycle and reduced proliferation is known to peak between 24 and 48 h after exposure to chemotherapy [19, 22, 23]. Collectively, this may account for the lack of difference between the CPT-11 and combination groups where tissue was collected at 72 h. Conversely, this lack in difference may also suggest that TLR4 downstream signalling may not play a major role in apoptosis seen in GIM. However, early time points coinciding with maximal protection from diarrhoea such as 24 h would need to be investigated to confirm both possibilities as it was observed in previous studies that apoptosis was decreased after 6 h in the colon [5, 19].

TLR4 signalling in the colon has been long associated with inflammatory conditions. As such, we next investigated TLR4-related transcripts known to play key roles in inflammatory responses. There were no differences in transcript expression of TLR4 and CD-14 in the colon between the groups. However, there was a decrease in expression of the co-receptor MD-2 in the colon of the IAXO-102 group compared to the vehicle group. These results are unexpected as previous studies have found increased TLR4 expression in the colon following chemotherapy [24, 25]. IL-6R and CXCR2 are both receptors associated with pro-inflammatory cytokines which are upregulated during inflammation [26, 27]. There was no difference between the groups in levels of CXCR2 expression, but a more interesting observation was the effect of the combination treatment on the levels of IL-6R transcript expression in the colon. A decrease in IL-6R levels was observed in the colon of the combination group compared to the CPT-11 group, which may indicate a mechanism by which IAXO is protective. IL-6 has been extensively studied in chemotherapy-induced GIM [27, 28] and in TLR4 knock-out mice lack an IL-6 response [5, 29]. Collectively, this eludes to IAXO–102 protecting against GIM through TLR4-depedent IL-6 regulation.

We also wanted to test whether TLR4 antagonism modulated CRC tumour growth and response to CPT-11 in our model. In the current study, IAXO-102 treatment alone led to a lower tumour volume compared to the vehicle group. A study by Pastille et al. has reported similar findings. They observed that by inhibiting TLR4 with an antagonist during intestinal inflammation, the development and progression of colonic tumours was significantly reduced compared to control mice [30]. They also observed a decrease in infiltration of pro-inflammatory cells and cytokines compared to control mice [30]. CPT-11 prevented tumour growth equally well in both groups. Based on the findings in the IAXO–102-alone group, it was expected that the combination group would have significant reduction in tumours compared to CPT-11 alone, but this was not observed. As such, there are clearly different roles for TLR4 during the development of tumours, compared to response to chemotherapy in our model. This is supported by other work showing conflicting roles of TLR4 in tumour response to cancer treatment [19, 21].

To explore the effect of TLR4 antagonsim on CRC tumours further, markers of cell proliferation and cell death were examined in all tumours at 72 h. Regarding levels of proliferation in the tumour, there were no differences between any of the groups, as such, the ability of IAXO–102 to decrease tumour growth is not attributable to increased cell turnover. As for levels of apoptosis, only the CPT-11 group had an increase in apoptosis levels compared to vehicle group. However, the results observed in both the proliferation and apoptosis scores were quite variable, which may be due to the heterogeneity of the tumour itself [31]. Consistent with the lack of significant effect of TLR4 antagonism on cell turnover, we were also unable to confirm any changes in inflammatory targets between the groups. Depending on where the tumour was examined, there may be differences in cellular morphology, gene expression, metabolism, and proliferation. This may be what caused the variability observed in the results and may have also affected the targeted treatments on these tumours.

While this is the first study to explore the specific TLR4 antagonist, IAXO–102, for its ability to protect against GIM in a CRC mouse model, there were limitations to the final interpretation of our findings. Statistical significance was difficult to establish in the RT-PCR analysis due to issues with the quality of cDNA which did not amplify the target genes as well as the housekeeper. Therefore, these results and numbers were not included in the analysis causing a decrease in sample size which led to difficulty in determining significance in the results. Whilst the advantages of using a tumour-bearing model means potentially more rapid translation into the clinical context, we need to also be mindful that tumours do create a systemic effect on the mice. So future studies could be undertaken in non-tumour-bearing mice with this compound to explore any other impacts.

Given IAXO-102 is a novel compound with inhibitory actions on TLR4, it would be important to look at any late side effects of IAXO-102 on the immune system that could be explored in future studies. Future work to confirm these findings will also need to include additional time points of tissue collection to look for changes coinciding with peak diarrhoea and weight change, as well as allowing longer growth trajectory of the tumours. Another limitation that needs to be noted in this study is that the diluent for IAXO-102 was not used as a vehicle. Components of the diluent included PEG and ethanol which may have impacted the results. However, the diluent for CPT-11 was prioritised as it was determined to be the more toxic diluent compared to the IAXO-102 diluent. Although the diluents used to reconstitute IAXO-102 can cause toxicity, it is only at high concentrations for extended periods of time [32–34]. These diluents have also been diluted with 45% saline solution which would decrease the concentration and, therefore, toxicity.

## Conclusion

In conclusion, the results demonstrated that IAXO-102 was able to attenuate CPT-11-induced diarrhoea as well as reduce tissue injury in the colon without impacting tumour response. However, given that there was no measurable impact on apoptosis or proliferation in either the colon or tumour, alternative mechanisms must account for these observations. Our work points to a downstream role for IL-6 in mediating the protective effects of IAXO-102, whereas other inflammatory markers were not significantly altered. Research efforts can therefore be shifted towards targeting IL-6R to understand its relationship with inflammation and apoptosis within the GIT.
